# Development of the lung

**DOI:** 10.1007/s00441-016-2545-0

**Published:** 2017-01-31

**Authors:** Johannes C. Schittny

**Affiliations:** Institute of Anatomy, University of Bern, Baltzerstrasse 2, CH-3012 Bern, Switzerland

**Keywords:** Lung development, Branching morphogenesis, Alveolarization, Microvascular maturation, Pulmonary acinus

## Abstract

To fulfill the task of gas exchange, the lung possesses a huge inner surface and a tree-like system of conducting airways ventilating the gas exchange area. During lung development, the conducting airways are formed first, followed by the formation and enlargement of the gas exchange area. The latter (alveolarization) continues until young adulthood. During organogenesis, the left and right lungs have their own anlage, an outpouching of the foregut. Each lung bud starts a repetitive process of outgrowth and branching (branching morphogenesis) that forms all of the future airways mainly during the pseudoglandular stage. During the canalicular stage, the differentiation of the epithelia becomes visible and the bronchioalveolar duct junction is formed. The location of this junction stays constant throughout life. Towards the end of the canalicular stage, the first gas exchange may take place and survival of prematurely born babies becomes possible. Ninety percent of the gas exchange surface area will be formed by alveolarization, a process where existing airspaces are subdivided by the formation of new walls (septa). This process requires a double-layered capillary network at the basis of the newly forming septum. However, in parallel to alveolarization, the double-layered capillary network of the immature septa fuses to a single-layered network resulting in an optimized setup for gas exchange. Alveolarization still continues, because, at sites where new septa are lifting off preexisting mature septa, the required second capillary layer will be formed instantly by angiogenesis. The latter confirms a lifelong ability of alveolarization, which is important for any kind of lung regeneration.

## Introduction

Regardless whether they call the land, the sky, or the water their home, all reptiles, birds and mammals rely on their lungs for gas exchange. The building blocks of a functional lung are:

- A tree of purely conducting airways (bronchi and bronchioles) transporting the air to and from the gas exchange region as well as many small trees of gas exchanging airways representing the acini (respiratory bronchioles and alveolar ducts). An acinus is defined as the unit that is served by the most distal purely conducting airways (terminal bronchioles). It represents the functional unit of the lung (Storey and Staub 1962; Tyler 1983).
- A very large surface area possessing a very thin air–blood barrier for gas exchange. It is located inside the acini (Gehr et al. 1978).
- An effective vascular system feeding the blood into the gas exchange area and bringing it in close contact to the air (Gehr et al. 1978; Hislop and Reid 1972).
- A surfactant system facilitating inflation stability, decreasing the work of breathing and contributing to the innate host defense of the lungs (Clements 1957; Sano and Kuroki 2005).

**Fig. 1:**
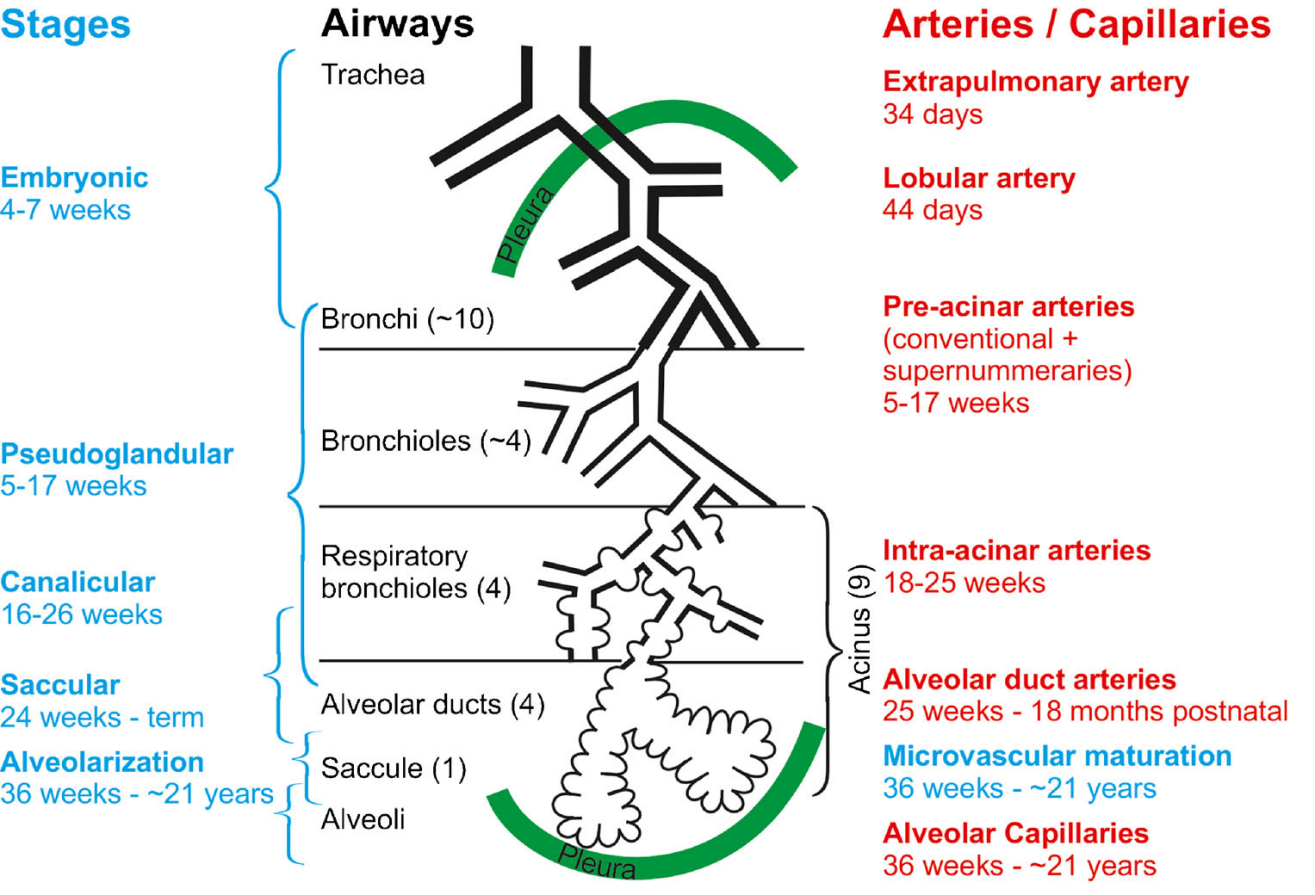
Development of the airways and arteries. The stages of lung development (*blue*) are correlated to the development of the airways (*black*) and the arteries (*red*). On average, an airway of a human lung ends in an alveolar saccule after 23 generations; however, due to the shape of the lung, a range of 18–30 generations has been observed. Pre-acinar arteries are formed out of a capillary plexus surrounding the growing lung buds (vasculogenesis). Intra-acinar arteries grow by angiogenesis (based on Hislop 2005, adapted from Schittny 2014 and by courtesy of Springer, Heidelberg)

Lung development is subdivided into three main periods (Table 1); the embryonic period, the fetal period and postnatal lung development. Lung organogenesis is part of the embryonal period. While fetal lung development consists in the pseudoglandular, canalicular and saccular stages, postnatal lung development comprises the stages of classical and continued alveolarization, as well as of microvascular maturation. The phases of lung development are mainly based on morphological criteria. Because most processes during lung development start proximal and extend into the periphery, all phases of lung development overlap (Fig. 1; Table 1).

**Table 1:** Stages of lung development and their time scale

Period	Stage	Duration	Characteristics
Embryonic	Embryonic	Rabbit: n.d.–E18 Sheep: E17–E30 Mouse: E9.5–E12 Rat: E11–E13 Monkey: n.d. – E55 Human: E26–E49 (4–7 weeks^a^)	Anlage of the two lungs; organogenesis; formation of major airways and pleura.
Fetal	Pseudoglandular	Rabbit: E18–E24 Sheep: E30–E85 Mouse: E12–E16.5 Rat: E13–E18.5 Monkey: E55 – E85 Human: E35–E119 (5–17 weeks)	Formation of bronchial tree and large parts of prospective respiratory parenchyma; birth of the acinus even if the acinar epithelia are not yet differentiated.
	Canalicular	Rabbit: E23–E27 Sheep: E80–E120 Mouse: E16.5–E17.5 Rat: E18.5–E20 Monkey: E75–E115 Human: E112–E182 (16–26 weeks^a^)	Formation of the most distal airways leading to completion of branching morphogenesis; first air-blood barrier; appearance of surfactant, acini are detectable due to epithelial differentiation.
	Saccular or terminal sac	Rabbit: E27–E30 Sheep: E110–E140 Mouse: E17.5–P4 Rat: E21–P4 Monkey E105–term Human: E168–E266 (24–38 weeks^a^)	Expansion of (future) airspaces.
Postnatal	Alveolarization, classical alveolarization (first phase)	Rabbit: E30–term (E31) Sheep: E120–term (E145) Mouse: P4 – P21 Rat: P4 – P21 Monkey: E125– <P180^b^ Human: E252 (36 weeks* preterm) – 3 years	Formation of secondary septa (septation) resulting in the formation of the alveoli; most of the alveolar septa are still immature and contain a double layered capillary network. Depending on the species alveolarization starts before or after birth.
	Alveolarization, continued alveolarization (second phase)	Rabbit: term (E31) – n.d. Sheep: term (E145) – n.d. Mouse: P14 – young adulthood (∼P36) Rat: P14 – young adulthood (∼P60) Monkey: < P180^b^– young adulthood (7–8 years) Human: 2 years – young adulthood (17–21 years)	Formation of secondary septa (septation) but now lifting off of mature alveolar septa containing a single layered capillary network.
	Microvascular maturation	Rabbit: n.d. Sheep: n.d. Mouse: P4 – young adulthood (∼P36) Rat: P14 – young adulthood (∼P60) Monkey: n.d. Human: ∼term – ∼3–21 years (timing uncertain)	Remodeling and maturation of interalveolar septa and of the capillary bed (the double layered capillary network is transformed to a single layered network). In a first approximation it takes place in parallel to alveolarization.

The stages are defined mainly by morphological criteria and their beginning and end do not represent sharp borders. In addition, stages are overlapping and regional differences are also common, especially between central and peripheral regions. Furthermore, litter size and nutrition influences the exact timing of development (Bryden et al. 1973; Burri 1999; Miettinen et al. 1997; Schittny et al. 1998, 2008; Ten Have-Opbroek 1991). Based on Schittny and Burri (2008) and Woods and Schittny (2016)

*Monkey* Rhesus monkey; *E* embryonic day (days post-coitum); *n.d*. not determined; *P* postnatal day

^a^Weeks post coitum

^b^Own unpublished observation

In a more general view, the inner surface of the lung is formed by two very different mechanisms, branching morphogenesis and septation (also called alveolarization) (Schittny and Burri 2008). The two lung buds (lung anlage) elongate and start a repetitive process of branching and of growth into the surrounding mesenchyme. Each branching generates a new generation of airways. Branching of epithelial tubes represents a very old developmental principle. It is also used during the development of glands (e.g., salivary and mammary glands) and the kidneys. All airways are formed by branching morphogenesis (Schittny and Burri 2008). Roughly 10% of the gas exchange surface area is formed by branching morphogenesis because, during the canalicular stage, the first future air–blood barriers are formed in the future alveolar ducts and saccules. The remaining ∼90% are formed by a completely different mechanism that is unique to lung development. The airspaces of the alveolar ducts and the respiratory bronchioles (if present) are subdivided by the formation of new inter-airspace walls called alveolar septa. This process is called septation for dividing airspaces by septa or alveolarization because the newly formed airspaces are called alveoli (Boyden and Tompsett 1965; Dubreuil et al. 1936; Engel 1953). The last step towards a mature gas exchange apparatus represents a thinning of the alveolar septa and a maturation of their microvasculature (stage of macrovascular maturation). Until recently, it was believed that this maturation takes place after alveolarization ceased (Burri 1975). However, the novel concept that alveolarization continues until young adulthood implies that alveolarization and microvascular maturation are parallel processes (Schittny et al. 2008).

This review mainly reports on human lung development, even if many of the mechanisms have been studied in animal models. All prenatal days and weeks are given as embryonic days (E), post-conceptional (p.c.) or gestational age, respectively. In clinical settings, the weeks of postmenstrual age is commonly used, which is equal to the gestational age plus 2 weeks.

## Embryonic period/organogenesis (weeks 4–7)

### Anlage of the lung and trachea

**Fig. 2:**
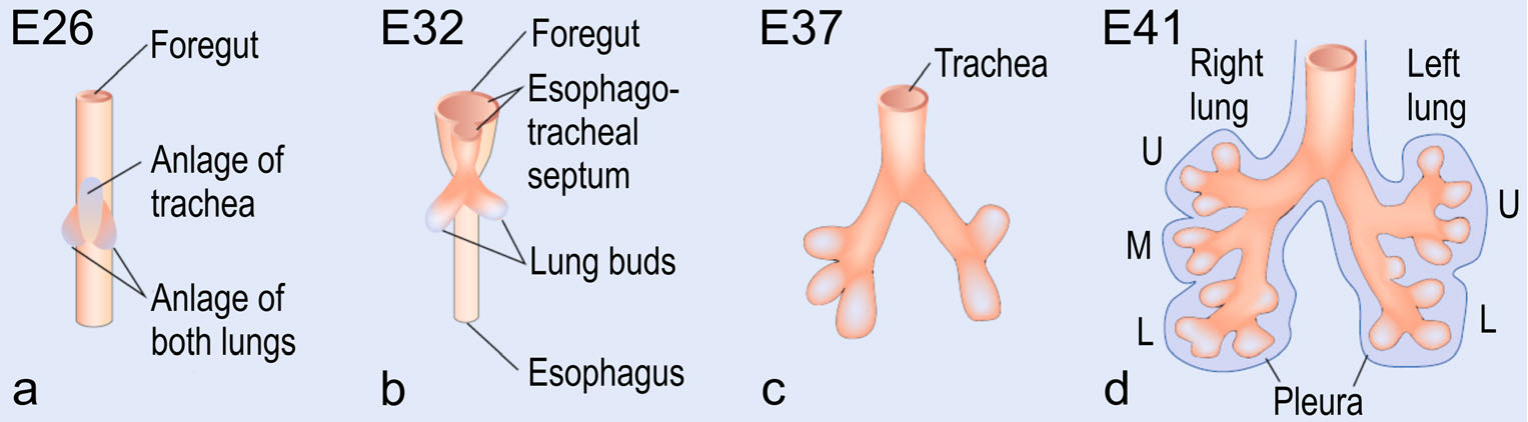
Early human lung development. At *E26*, post-conceptional, the anlage of the two lungs forms by outpouchings of the foregut on both sides lateral of the anlage of the trachea (**a**) (Cardoso and Lu 2006). The prospective trachea forms by a distal-to-proximal segregation from the foregut. At *E32*, the two lung anlages give rise to the two future main bronchi (**b**). Due to continued branching, the lobar bronchi are formed at *E37* (**c**). Later, at *E41*, the segmental bronchi follow (**d**). Organogenesis is completed after the formation of the pleura (**d**). *U* upper lobe; *m* middle lobe; *l* lower lobe (from Schittny 2014, by courtesy of Springer, Heidelberg)

In humans at day 26 p.c., the anlage of the right and left lungs appears as two independent outpouchings of the ventral wall of the primitive foregut The two lung buds are located right and left of the anlage of the trachea (Cardoso and Lu 2006). They are *not* the result of the first branching of a common lung bud as postulated earlier (Fig. 2a). Both lung buds begin elongating and start a repetitive circle of growth into the surrounding mesenchyme and branching (branching morphogenesis; Fig. 2b–d; Table 1).

The primitive foregut divides into the esophagus and trachea after a deepening and joining of the laryngotracheal sulci of the lateral walls of the foregut. Mesenchymal cells surrounding the forming trachea are condensing focally and differentiate into precursors of cartilage towards the end of the embryonic period. With further development of the bronchial tree, the formation of the cartilage moves distally until it reaches the smallest bronchi (25 weeks).

During weeks 5–7, the visceral pleura forms as a product of the splanchnic mesoderm. In parallel, the parietal pleura forms out of the somatic mesoderm layer covering the inner surface of the body wall. Thereafter, the visceral pleura starts to fold into the lung separating the tissues surrounding the lobar bronchi. It forms the lobar fissure separating the lung lobes. In parallel with the formation of the pleura, the pleuropericadial folds meet and fuse with the foregut mesenchyme. Caudally at the posterior body wall, the two pleuroperitoneal membranes start growing towards the posterior edge of the septum transversum. All of them meet and fuse, resulting in a closure of the pleural cavities (Sadler 2014; Schoenwolf et al. 2015).

### Epithelial–mesenchymal interactions

Because the anlage of the lungs forms out of the primitive foregut, the future lung epithelia are of endodermal descent. The mesoderm, where the epithelial tubes push in, is obviously of mesodermal descent. The dual descent of the lung tissue is of importance for the branching morphogenesis, because branching is governed by an intensive cross-talk between epithelial and mesenchymal cells and the factors they are producing. The epithelial cells are supported by a basement membrane and surrounded by an extracellular matrix that is in large parts produced by the mesenchymal cells. The components of the extracellular matrix including the basement membrane are different at the terminal bud, the branching points and in the more proximal parts of the bronchial tree where epithelial differentiation already started (Schittny and Burri 2004). A very specific differential expression of factors like fibroblast growth factor 10 (FGF-10), bone morphogenic protein 4 (BMP-4), Sonic Hedgehog (Shh), retinoic acid, Notch, TGF-β and others give the instructions for the branching morphogenesis. During this process, the epithelial tubes go through repetitive circles of branching and outgrowth into the surrounding mesenchyme (for reviews, see Cardoso and Lu 2006; Hines and Sun 2014; Swarr and Morrisey 2015).

### Vasculogenesis of pulmonary circulation

The pulmonary arteries are running in parallel to the airways, delivering the blood to the capillary network of the alveoli. The pulmonary veins collect the blood and run inter-axially at the surface of the pulmonary units, be it segments, sub-segments or as the smallest unit the acini. The veins are embedded in the inter-axial connective tissue. In order to reach the hilum, the large venous branches join the arteries and airways in the most central areas (Verbeken et al. 1996). The first pulmonary vessels are formed as a plexus in the mesenchyme surrounding the lung buds by vasculogenesis—the de novo formation of vessels due to a differentiation of mesenchymal cells. The plexus is cranially connected to the aortic sac and caudally to the left atrium. During branching of the future airways, a new capillary plexus is formed as a halo surrounding each newly formed bud. Each plexus adds to the future pulmonary circulation. The final structure is achieved by intussusceptive remodeling, pruning and angiogenesis of the primary formed vessels. Thus, the forming bronchial tree of airways serves as a template for the vascular tree of blood vessels and lymphatic drainage (Burri 1985; Djonov and Burri 2006).

### Clinical aspects of the embryonic period

Malformations during organogenesis are often related to lung bud formation and development, tracheal/esophageal separation and an incomplete formation of the diaphragm. As for any other organ, these abnormalities show a high mortality and postnatal morbidity. Structural malformations like pulmonary agenesis or aplasia, as well as pulmonary valve stenosis are generally viewed as nonsurvivable, whereas others show variable degrees of morbidity. An incomplete closure of the pericardial–peritoneal canals causes a diaphragmatic hernia and a compression of the developing lung resulting in pulmonary hypoplasia (Kinane 2007). A variety of permutations of abnormal connections between the trachea and the esophagus represent a common congenital malformation during pulmonary organogenesis. It includes tracheaoesophageal fistula and esophageal atresia, a defect where the upper and lower segments of the esophagus do not connect. In full-term infants, the postsurgical survival is high but in low-birth-weight babies, these malformations are often accompanied by other congenital defects resulting in a higher risk for poor outcomes (Kovesi and Rubin 2004).

## Pseudoglandular stage (weeks 5–17)

### Formation of the bronchial tree

**Fig. 3:**
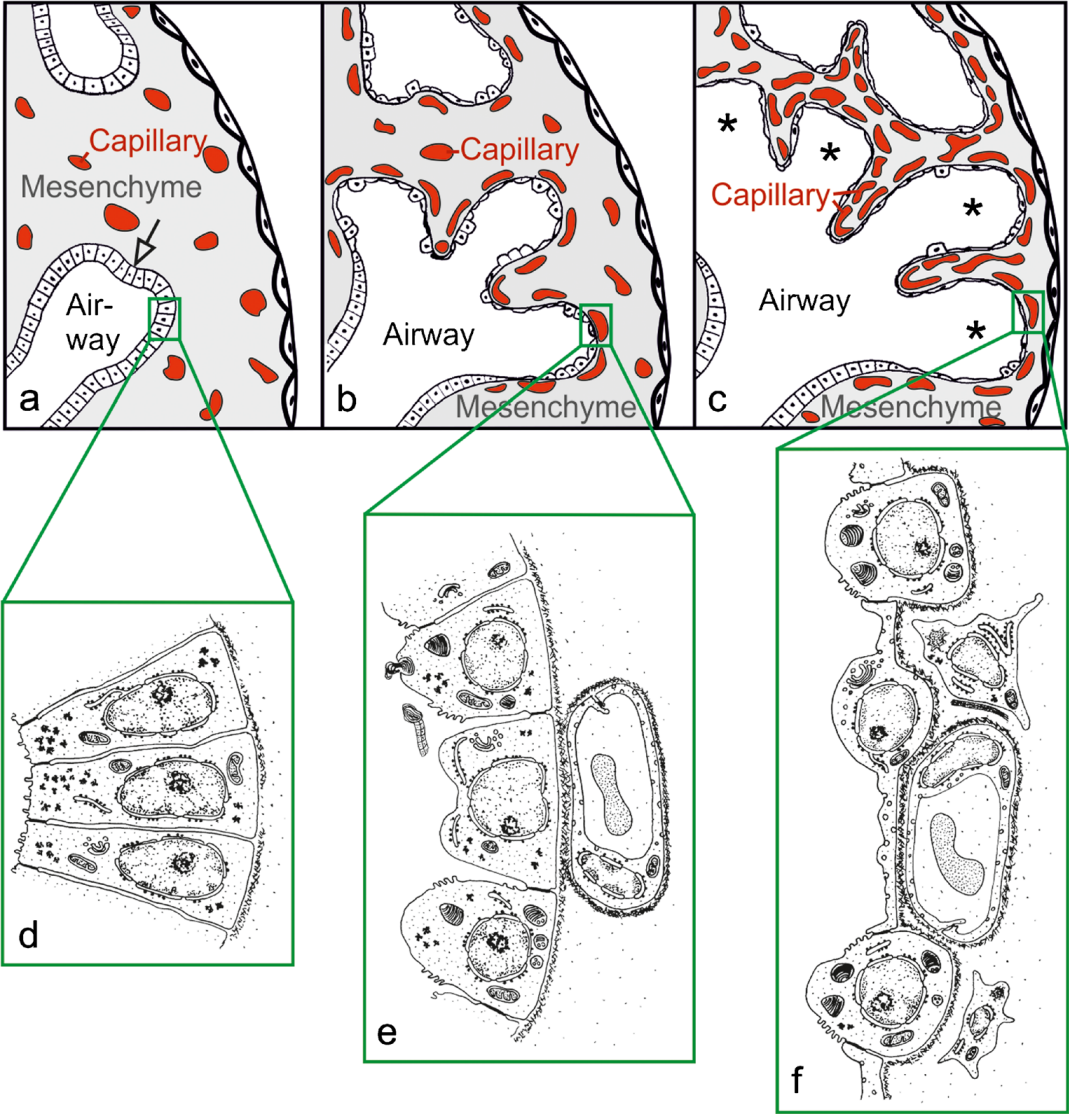
Morphological development of the lung parenchyma during the pseudoglandular, canalicular and saccular stage. The epithelial tubules branch repeatedly during the pseudoglandular stage and penetrate into the surrounding mesenchyme (**a**, *open arrow*, branching point). The mesenchyme contains a loose capillary network (**a**). The epithelium itself is tall and columnar (**d**). The canalicular stage (**b**) is characterized (1) by a differentiation of the epithelial cells into type I and type II epithelial cells (**e **, **f**), (2) by a widening of the future airways (**b**), (3) by a multiplication of the capillaries and their first close contacts to the epithelium (**b**) and (4) by the formation of first future air–blood barriers (**e**→**f**). During the saccular stage (**c**), thick immature inter-airspace septa are formed due to a further condensation of the mesenchyme. The immature septa contain a double-layered capillary network, one layer on either side of the septum. The terminal ends of the bronchial tree represent wide spaces and are called saccules (*asterisks*). (**a**–**c** modified from Caduff et al. 1986; **d**, **e** modified from Burri and Weibel 1977, **f** from Woods and Schittny 2016, by courtesy of Cambridge University Press, New York)

During the formation of the bronchial tree the lung looks like a tubular gland, which gives this stage its name. Outgrowth of the terminal bud into the surrounding mesenchyme is followed by branching and formation of the next generation of future airways. The future airways are loosely embedded in the mesenchyme (Fig. 3a). During the pseudoglandular stage, approximately the first 20 generations of the future airways are formed in humans (Kitaoka et al. 1996). Therefore, by the end of this stage, the first few generations of alveolar ducts are already laid down. Solid epithelial sprouts start to form in the walls of trachea and bronchi and invade the mesenchyme similar to the outgrowth of the terminal bud of the bronchial tree. These sprouts give rise to the future mucous glands that are first found at weeks 12–14 in humans (Burri 1985).

### Epithelial and smooth muscle cell differentiation

Starting proximally, the future airways are lined by a very high columnar epithelium (Fig. 3d). The height of the epithelial cells decreases continuously toward the terminal branches where cuboidal epithelium is found. The cuboidal epithelia of the terminal buds maintain their undifferentiated state until branching morphogenesis is completed during the canalicular stage. In some species like human, branching morphogenesis may even continue until the saccular stage. During the pseudoglandular stage, the first ciliated, goblet and basal cells appear in the proximal airways. Thereafter, epithelial differentiation progresses into the periphery (Burri 1985).

A continuous layer of α-smooth muscle actin-positive cell starts to form around the most proximal future airways. The layer becomes discontinuous in the more distal parts of the bronchial tree and ends in front of the terminal buds. These contractile cells start to perform spontaneous contractions pushing peristaltic waves of inter-bronchial fluid into the periphery, causing a rhythmic extension of the distal airways including the terminal buds. It was postulated that these movements stimulate branching morphogenesis and prevent uncontrolled expansion of the airways as pulmonary fluid is secreted into the lung (Schittny et al. 2000; Sparrow et al. 1994).

### Arteries and veins

During the pseudoglandular stage, the arteries and veins continue to develop as already described in the previous sections (organogenesis). However, the arterial tree contains a higher number of generations than the bronchial tree. On average, there are 20% additional branches in the arterial tree versus the bronchial tree. These so-called “supernumerary” arteries do not strictly follow the same bronchial tree as the conventional ones but split off at right angles. Usually they serve the “recurrent” gas exchange tissue adjacent to the larger airways (Hislop and Reid 1973; Hislop 2002).

### Clinical aspects of the pseudoglandular stage

During the pseudoglandular stage, lung development depends on mechanical stimuli. The spontaneous contractions of the airways have already been discussed above. Around 10 weeks p.c., fetal breathing movements start in humans. These movements cause additional stretching of the lung tissue and move fluid in and out of the lungs (Koos and Rajaee 2014). Recent studies have demonstrated that mechanical stimuli upregulate the release of serotonin via mechanosensitive channels promoting differentiation of epithelial cells (Pan et al. 2006). In addition, stretching stimulates epithelial cell proliferation (Liu et al. 1999) and increases the secretion of surfactant lipids by type II epithelial cells (Scott et al. 1993).

A congenital diaphragmatic hernia leads to pulmonary hypoplasia and hypertension if an ascension of the abdominal organs into the chest cavity and a compression of the lung(s) takes place. The compression leads to a reduction of the bronchial generations and later to a reduction of the gas exchange surface area. These conditions lead to a high rate of mortality (Kitagawa et al. 1971). An elevation of the inter-pulmonary pressure by an occlusion of the trachea improved the outcome in animal experiments. However, side effects prevented this measure being applied clinically (Vuckovic et al. 2013; Wilson et al. 1993).

A prolonged imbalance of the pressure between the extra-luminal space and the inter-luminal airway has lasting negative effects on lung development. These conditions are encountered due to a fetal exposure to an oligohydramnios. It causes pulmonary hypoplasia and an 80% higher risk of respiratory failure as compared to unaffected fetuses (Chien et al. 2014). An oligohydramnios is often part of an autosomal recessive polycystic kidney disease. Because kidney development also includes branching morphogenesis, the pulmonary hypoplasia may be caused by a combination of the genetic defect and the compression of the developing lung (Hartung and Guay-Woodford 2014).

## Canalicular stage (weeks 16–26)

The canalicular stage comprises the differentiation of the epithelia that allows the morphological distinction between conducting and respiratory airways. This distinction permits the recognition of the acinus/ventilatory unit for the first time (Boyden 1971). However, most of the acinar airways are already formed during the pseudoglandular stage and only the very distal generations are formed during the canalicular stage (Kitaoka et al. 1996). Furthermore, the alveolar epithelium comes into close contact with the mesenchymal capillary network in order to form the first future air–blood barriers.

### “Canalization” of the lung parenchyma

At the end of the saccular stage, the mesenchyme located between the future airways contains a loose three-dimensional vascular network. This network starts to proliferate due to intensive angiogenesis resulting in a high capillary density. The future airways that will transform to alveolar ducts start to grow in width and length, change their shape and appear like “canaliculi” (Fig. 3a→b). The canalization of the mesenchyme by respiratory airways and capillaries gave this stage its name. The growth of the airways causes a condensation of the mesenchyme (Fig. 3a→b). Programmed cell death (apoptosis) contributes to the condensation of the mesenchyme, where not only the volume but also the total number of mesenchymal cells is reduced (Rogelj et al. 1989).

### Epithelial differentiation: formation of the air–blood barrier

**Fig. 4:**
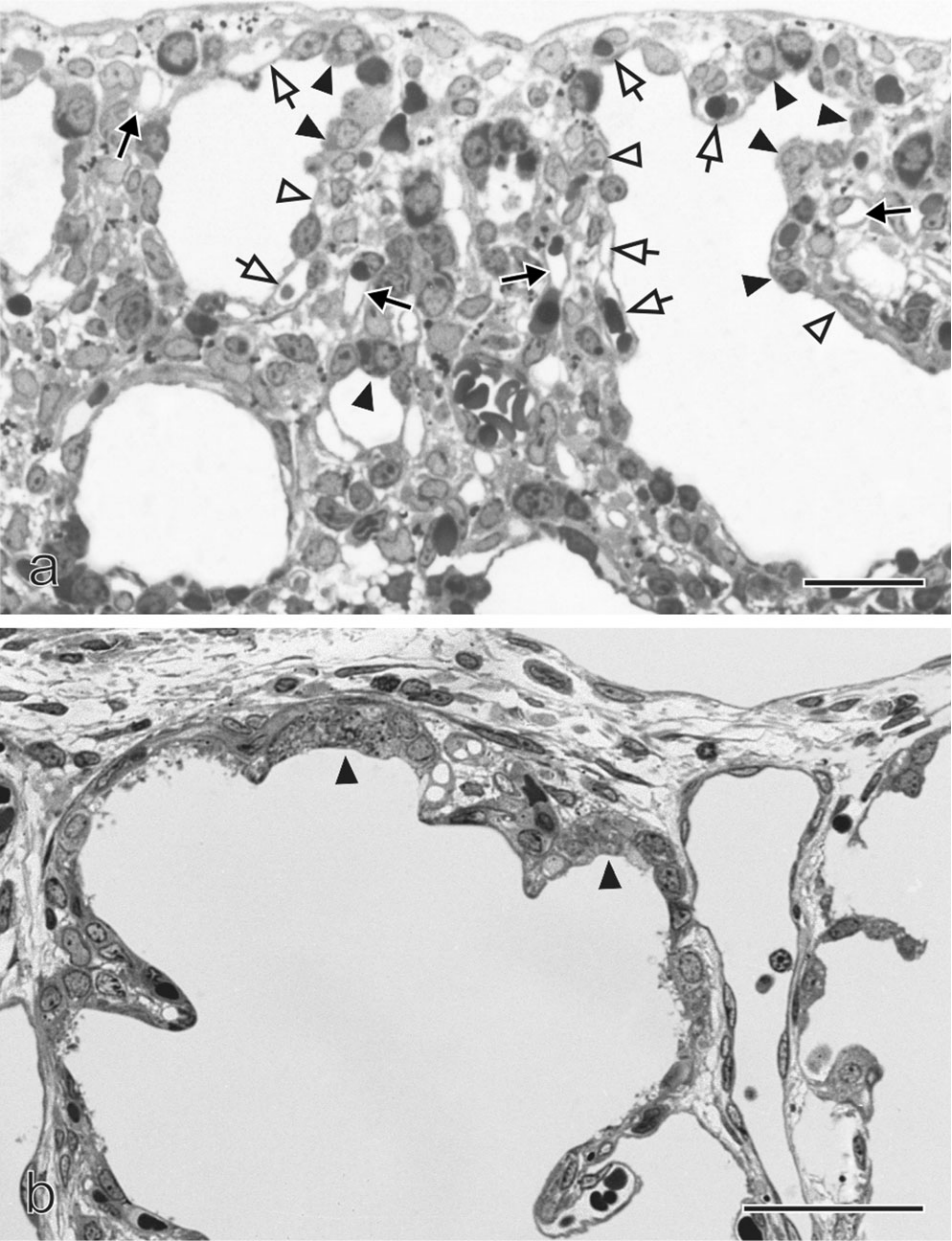
Formation of the air–blood barrier. During the early canalicular stage (**a**, rat lung) the epithelium of the terminal airways is still cuboidal and glycogen-rich (*closed arrowhead*). Already a bit more proximal, the epithelium begins to flatten out (*open arrowhead*) and starts to form the first optimized future air–blood barriers. During the latter process, the capillaries of the mesenchyme (*closed arrow*) “move” towards the epithelium (*open arrow*). In humans (**b**), remnants of the cuboidal epithelium (*closed arrowhead*) are still present at the uttermost periphery of the gas exchange region at postnatal day 26, even if alveolarization had already started approximately 6 weeks earlier. This finding illustrates the large overlap between different phases of lung development, especially if peripheral and central parts are compared. Light microscopical images, *bar* 50 μm. (From Woods and Schittny 2016, by courtesy of Cambridge University Press, New York)

The undifferentiated glycogen-rich cuboidal epithelial cells of the future alveolar ducts differentiate into type I and type II alveolar epithelial cells (Fig. 3e→f). However, even if the epithelial differentiation becomes visible during this stage, the fate of the cells is decided much earlier (Cardoso and Lu 2006). The type I cells form thin sheet-like extensions covering most of the inner surface area of the alveolar ducts and sacculi. The type II cells are located between the type I cells, often close to the locations where three alveolar septa meet. At these locations, where the endothelium of the capillaries comes into close contact with the type I alveolar epithelial cells, the future air–blood barrier forms (Figs 3b, e→f, 4a). At their contact surface, both cell types form a very thin sheetlike structure and are separated by a common, three-layered basement membrane. The latter is formed by fusion of the endothelial and epithelial basement membranes. It possesses two lamina lucida facing the epithelial or endothelial cells, respectively and one fused lamina densa in the center of the basement membrane (Figs. 3f, 4a, b).

Currently, the formation of the air–blood barrier is poorly understood. It is likely that an intensive “cross-talk” between the endodermally-derived epithelium and the mesodermally-derived endothelium takes place. This hypothesis is supported by observations made in a transgenic mouse model where the sequence coding for the nitrogen-binding site of laminin (γ1III4, within the laminin-γ1-chain) was selectively deleted. The basement membranes of the air–blood barrier are missing or disrupted in a large part in these mice. The mice die neonatally due to a failure of pulmonary gas exchange (Willem et al. 2002).

Shortly after their appearance, type II epithelial cells start to produce surfactant. The accumulation of surfactant becomes morphologically visible by the intracellular formation of lamellar bodies. In most species, surfactant appears at about 80–90% of the total duration of the gestational period. In humans, surfactant appears earlier. Small amounts are already present at 60% of gestation (weeks 22–24) (Burri 1999). At first, surfactant appears to be less abundant in basal lung regions than in apical ones (Howatt et al. 1965). This finding may explain clinical observations that in preterm infants hyaline membrane disease is more pronounced in basal than in apical lung regions.

Type II alveolar epithelial cells represent the progenitor of type I epithelial cells (Bachofen and Weibel 1977). Therefore, it is not surprising that the future alveolar epithelial cells contain a few, small, lamellated bodies before differentiation into type I and type II cells becomes morphologically visible (Mercurio and Rhodin 1976).

### Pulmonary acinus

At the bronchioalveolar duct junction (BADJ), an abrupt change of the epithelia from ciliated and Club cells (originally called Clara cells; Winkelmann and Noack 2010) to type I and type II alveolar epithelial cells takes place. This junction is formed during the canalicular stage when epithelial differentiation occurs and is of particular importance because it represents a stem cells niche (McQualter et al. 2010; Nolen-Walston et al. 2008). Very recently, it was shown by the group of the author that the bronchioalveolar junction stays constant throughout lung development at the location or better at the generation of the airways, where it was originally formed (Barre et al. 2014, 2016). In animals like mice and rats that do not possess respiratory bronchioles, the bronchioalveolar duct junction represents the entrance to the acinus (Tyler 1983). Therefore, in these animals, the entrance of the acini also stays constant and, as a consequence, the number of acini is first defined during the canalicular stage and stays constant throughout lung development into adulthood. Furthermore, the growth of the lung parenchyma in volume and surface area takes place exclusively inside the acini without increasing their number (Barre et al. 2016).

Pulmonary acini are defined as the small trees of gas exchanging airways that are ventilated by the most distal purely conducting airways (terminal bronchioles). In mammals like humans that possess respiratory bronchioles, the acinus starts approximately 3–4 generations proximal of the bronchioalveolar duct junction and ends ∼4 generations of alveolar ducts distal of the bronchioalveolar duct junction (Tyler 1983). The region of the alveolar ducts is called the ventilatory unit (Storey and Staub 1962) and represents the corresponding region of the rodent acinus. Even if it was not measured in humans, it may be hypothesized that the number of ventilatory units stays constant after they have been formed during the canalicular stage. The future respiratory bronchioli are formed during the pseudoglandular stage but they are not detectable until their alveoli are formed much later during alveolarization. Therefore, during lung development, the entrance of an acinus should be defined as its most proximal *future* respiratory bronchiole. Based on this definition, the number of acini also stays constant in humans, even if it takes until the start of alveolarization that they become visible (Barre et al. 2016).

**Fig. 5:**
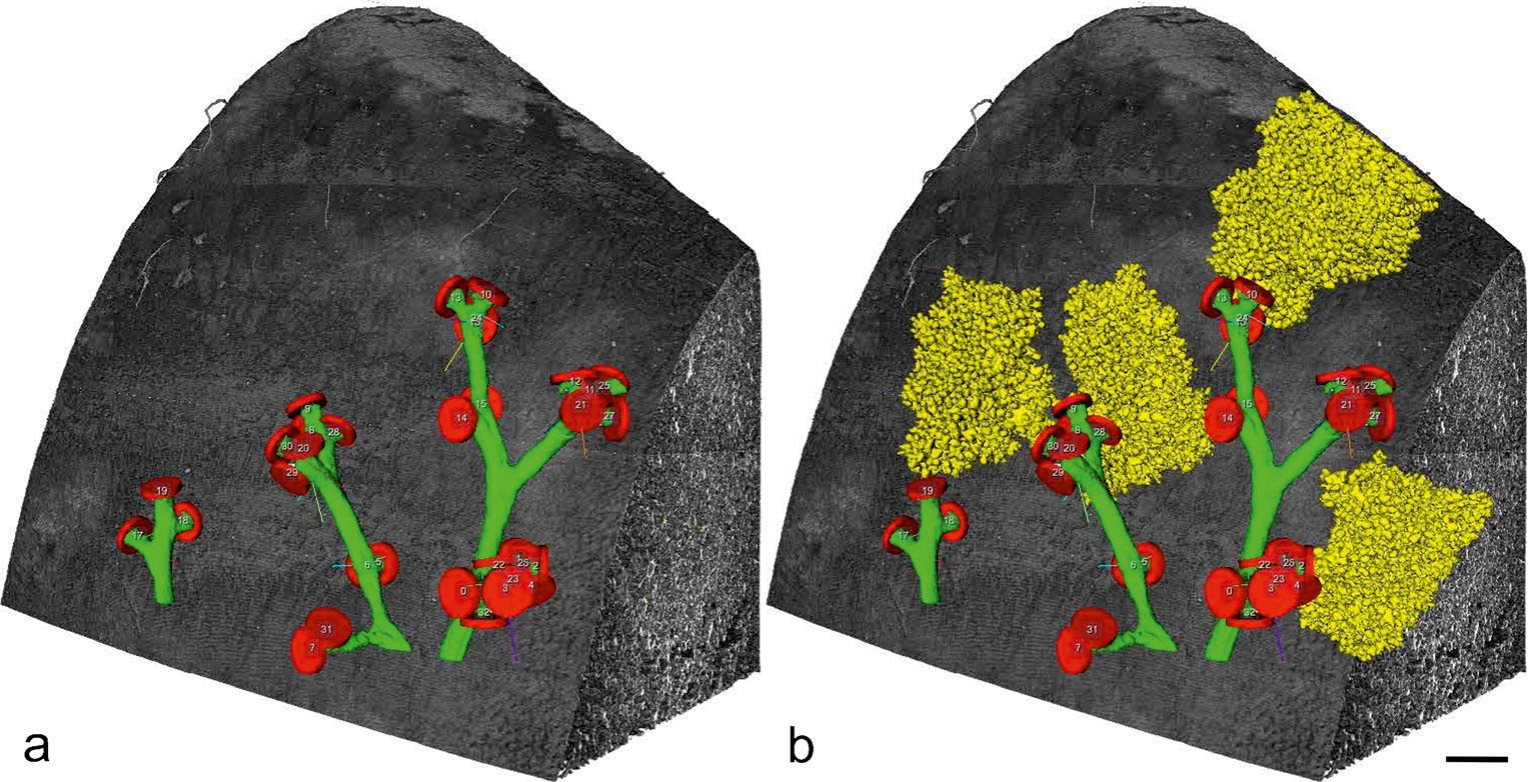
3D visualization of distal conducting airways and acini of a rat lung. Distal conducting airways (bronchioles) are shown in *green*. The bronchioalveolar duct junction is labeled with a *red disk*. The disks were used as segmentation stoppers in order to separate the conducting from the acinar airways (*yellow*). The lung tissue is shown in shades of* gray*.* Left panel* conducting airways and segmentation stopper;* right panel* four acini are shown in addition to the structures shown in the l*eft panel*;* bar* 0.5 mm. (From Haberthur et al. 2013, by courtesy of The American Physiological Society, Bethesda, MD, USA)

Using synchrotron radiation-based X-ray tomographic microscopy, rat acini were visualized in 3D (Fig. 5) (Haberthur et al. 2013). On average, the rat acini start off at postnatal day 4 as a complex arrangement of ∼30 segments of alveolar ducts, ∼150 saccular airspaces and a volume of 0.11 μl (mm^3^). Hence, our current view that all of these 150 saccular airspaces are formed by branching morphogenesis is in contradiction to the low number of segments of alveolar ducts. Further studies based on 3D images are necessary to resolve this contradictory result. At young adulthood (day 60), a rat acinus still has ∼30 segments of alveolar ducts but the number of airspaces (now alveoli) increased to ∼3500 and the volume to 1.2 μl (Barre et al. 2014; Tschanz et al. 2014). All the acini are arranged to be space filling, in terms of their contact to each other, as well as in terms of the internal arrangement of the ducts and alveoli.

**Fig. 6:**
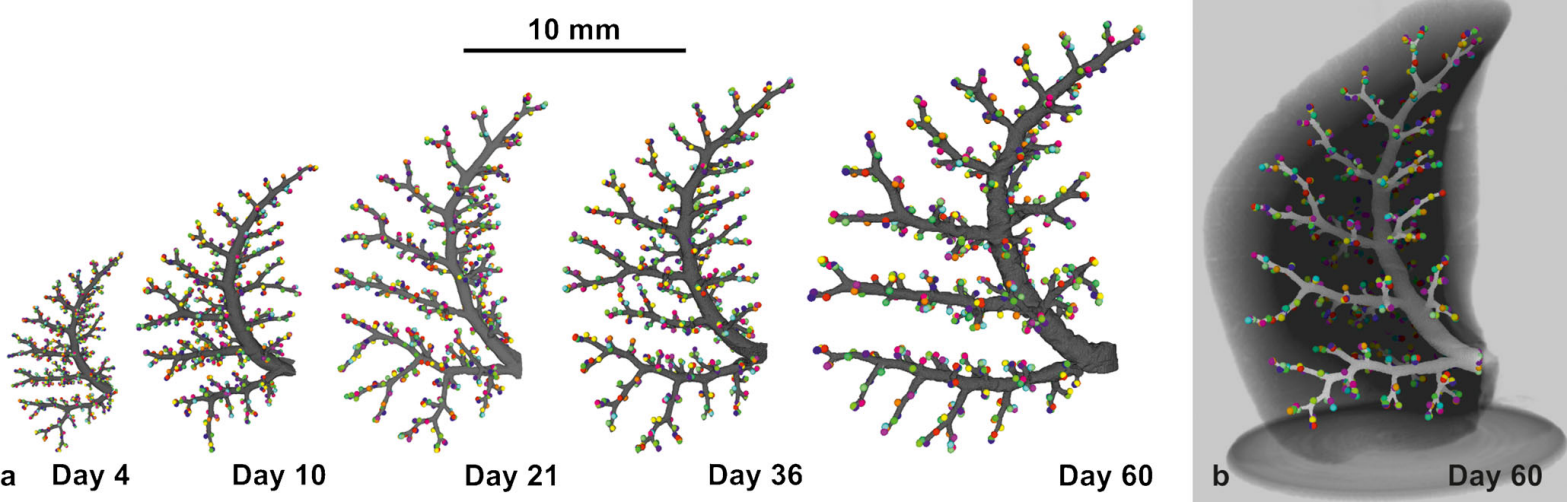
Development of the bronchial tree of the conducting airways of the right middle rat lung lobe. The walls of the conducting airways are shown in *gray*. The *colored spheres* represent the entrances of the acini and were used as segmentation stopper. These three-dimensional visualizations show the large similarity of the conducting airways structure at *Days 4*,* 10*,* 21*,* 36* and* 60* and between different individuals (**a**). **b** The bronchial tree at *Day 60* embedded in the surrounding lung parenchyma. Due to the monopodial branching pattern of the rat airways, the acinar entrances are not evenly distributed inside any lung lobe. Most of the acinar entrances are located on a virtual cylinder around the main airways and the most peripheral ones facing the pleura with their distal part. As a consequence an outer cortex exists that is free of acinar entrances (altered from Barre et al. 2016, by courtesy of The American Physiological Society, Bethesda, MD, USA)

Furthermore, the development of the conducting airways of rats was also visualized (Fig. 6) (Barre et al. 2016). Hence, these airways show a *monopodial* branching pattern. The acinar entrances are facing to the primary, secondary and tertiary bronchi and the acini themselves form a cylinder around the major airways. The entrances of the most peripheral acini (acini that are located directly underneath the pleura) are all directed to the center of the lung. Therefore, a cortex of the lung forms, which is free of acinar entrances and consists exclusively of lung parenchyma (Figs. 5, 6) (Haberthur et al. 2013). It has been shown by Kizhakke Puliyakote et al. (2016) that the cortical acini are larger than the central ones. Comparing the tree of conducting airways, a very high similarity between the different days is eye-catching, even if different subjects were used for each day (Fig. 6).

### Clinical aspects of the canalicular stage

Reaching the canalicular stage is very important for very immature and prematurely born infants. At this time point, first future air–blood barriers are formed and at least a minimal production of surfactant is produced. A survival is feasible but only with specialized support of a neonatal intensive care unit. It is mostly dependent on the amount of available gas exchange surface area in the neonatal lung. First, the air–blood barriers have to be formed at that point and, second, the lung has to be inflated. The latter mostly depends on the amount of available surfactant. Unwanted side effects of the intensive treatment are correlating with the immaturity of the lungs and may cause a chronic lung disease called bronchopulmonary dysplasia (see below).

In patients with alveolar capillary dysplasia (ACD), the alveolar epithelium is surrounded by mesenchyme containing small blood vessels instead of capillaries. In addition, ACD is often accompanied by a misalignment of the pulmonary veins. The malformation results in a reduced capillary density, reduced air–blood interface, thickened alveolar septa and pulmonary hypertension. Vascular endothelial growth factor (VEGF) and endothelial nitric oxide synthase (eNOS) are linked to this lethal congenital lung abnormality (Groenman et al. 2005; Kool et al. 2014).

## Saccular stage (weeks 24–38)

The saccular stage represents an intermediate stage, when branching morphogenesis ceases and alveolarization has yet to start. This intermediate stage may be necessary because branching morphogenesis and alveolarization are completely different genetic programs that apparently do not take place in parallel (Cardoso and Lu 2006; Morrisey et al. 2013). Most likely, the most distal generations of the airways were already formed at the end of the canalicular stage. However, depending on the species, very few terminal airways may also be formed during the saccular stage by branching morphogenesis (Woods and Schittny 2016).

### Expansion of the gas-exchange area

At the beginning of the saccular stage, the terminal (acinar) airways are growing in length and are widening. They form clusters of larger airspaces. Especially, the terminal ends are widening and form so-called sacculi, which give their name to this stage (Fig. 3c). The enlargement of the future gas exchange region causes a condensation of the mesenchyme. At locations where two airspaces meet, primary septa are formed. The capillary networks stay very close to the septal surfaces and are separated by a central layer of condensed mesenchyme forming a core of connective tissue. As a result, the primary septa contain a double-layered capillary network. Most of the surface of the primary septa is covered by type I epithelial cells. The remaining surface is filled in by type II epithelial cells that produce surfactant and serve as progenitor cells for the type I epithelial cells (Fig. 3f). During this stage, smooth muscle cell precursors move in and start to lay down a fibrous network of elastic fiber and collagen fibrils that represent a prerequisite for the following alveolarization.

### Time point of birth

The stage of lung development at term correlates with the physical activity of the newborn. The marsupial quokka wallaby (*Setonix brachyurus*) is born in the canalicular stage and may be the least physically mature mammal directly after birth (Makanya et al. 2001). Insessorial mammals like mice and rats are born during the saccular stage (Amy et al. 1977; Burri 1974). In precocial mammals like sheep (Alcorn et al. 1981) and guinea pigs (Sosenko and Frank 1987), alveolarization starts well before birth and at term their lungs appear nearly mature. The physical activities of human newborns are in between insessorial and precocial mammals and therefore at term human lung development is in its early alveolar stage (Zeltner et al. 1987). Regardless of the stage of lung development at term, alveolarization continues at least as long as the lungs are growing.

### Clinical aspects of the saccular stage

Very early prematurely born infants have to use their lungs for gas exchange at the end of the canalicular stage and during the saccular stage. As a life-saving measure, many of them receive surfactant, steroids, or oxygen and are mechanically ventilated, causing damage at the cellular level due to oxygen toxicity and volutrauma. In addition, these infants have to cope with pulmonary inflammation and a steroid-induced re-programming lung development. As a result, alveolarization does not progress as well as in term-born infants. The resulting disease is called bronchopulmonary dysplasia (BPD). Infants showing BPD have fever and therefore larger alveoli, insufficient vascularization and airflow limitations. All of these conditions cause a reduction of gas exchange in its own right. BPD-induced alterations of the lungs often persist into adulthood or throughout the whole life (Caskey et al. 2016; Madurga et al. 2013).

## Alveolarization (week 36–young adulthood)

**Fig. 7:**
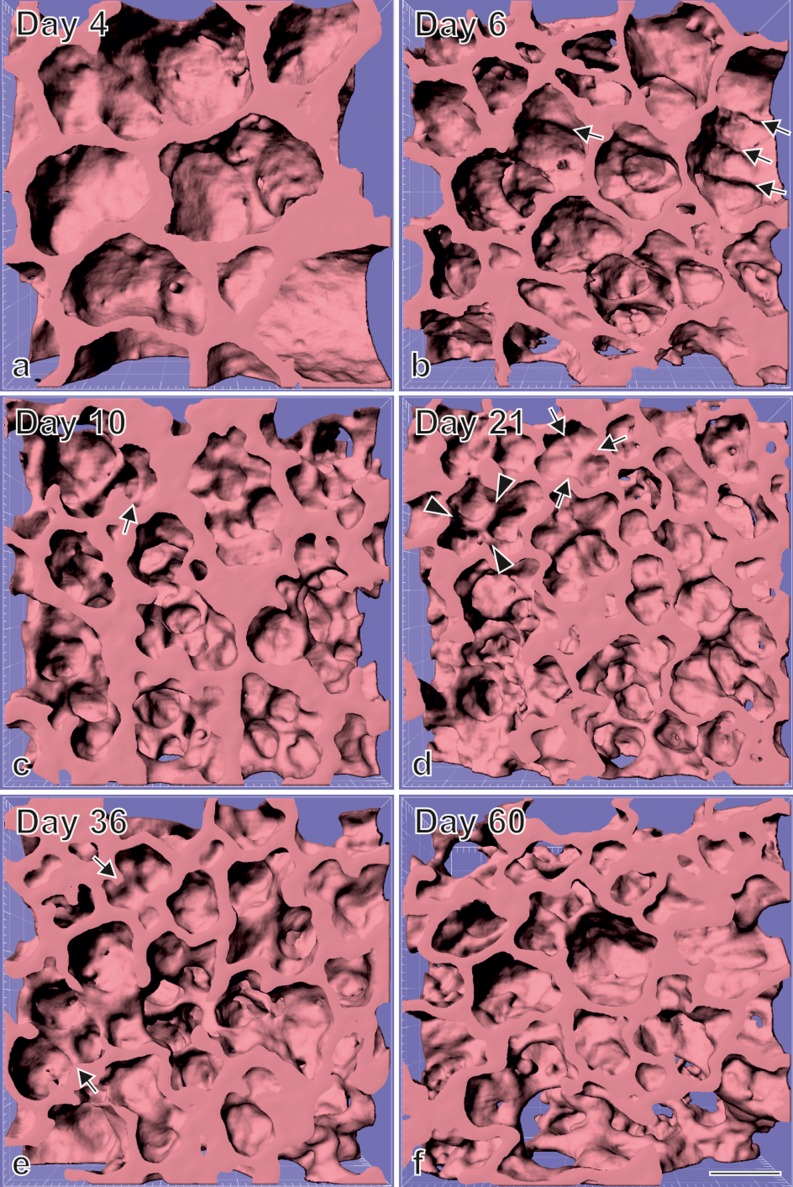
Visualization of alveolarization. 3D visualizations of terminal air spaces were obtained during postnatal rat lung development. At postnatal day 4 (**a**), large saccules are observed. They were formed by branching morphogenesis. Between days 6 and 36 (**a**–**e**), many low-rising septa are present, which are indicative for newly-forming septa (*arrows*). On the same days, higher-rising, mature septa are also visible (*arrowheads*), while between days 4 and 21 (**a**–**d**), the size of the terminal airspaces decreases, with an increase of the airspaces observed between days 21 and 60 (**e **, f). Synchrotron radiation-based X-ray tomographic microscopy was applied for 3D imaging after a classical embedding for electron microscopy. *Bar* 50 μm. Due to the perspective view, the *bar* is only correct at the surface of the sample. (From Mund et al. 2008, by courtesy of Springer, Heidelberg)
- In the classical model of alveolarization, it was postulated that alveolarization ceases after the capillary layers in the alveolar septa mature during microvascular maturation (Burri et al. 1974). Using recently developed techniques, it was shown for rabbits (Kovar et al. 2002), rhesus monkey (Hyde et al. 2007), rats (Schittny et al. 2008), mice (Mund et al. 2008) and humans (Herring et al. 2014; Narayanan et al. 2012) that alveolarization continues at least as long as the lungs are growing.
- New alveolar septa may be lifted off both immature and mature alveolar septa. Therefore, new septa or new alveoli, respectively, may be formed principally at any time, even during adulthood. Two phases of alveolarization are distinguished: classical (or bulk) alveolarization (Fig. 8a–d) and continued alveolarization (Figs. 8e–f, 9) (Schittny et al. 2008; Tschanz et al. 2014; Woods and Schittny 2016). However, the rate of increase in the number of alveoli is smaller during continued alveolarization than during classical alveolarization (Hyde et al. 2007; Tschanz et al. 2014).

During the past ∼10 years, new tools like counting the number of alveoli (Hyde et al. 2004), estimating the length of the free septal edge and the formation of new septa (Schittny et al. 2008), as well as high-resolution tomographic imaging of alveoli, acini and the alveolar microvasculature (Lovric et al. 2013; Schittny et al. 2008; Stampanoni et al. 2010; Vasilescu et al. 2012) and ^3^Helium Magnetic Resonance Imaging (^3^HeMRI) have become available. ^3^HeMRI is used for the measurement of diffusion distances, which allows the estimating of the size of the airspaces (Woods et al. 2006; Yablonskiy et al. 2009). Applying these new tools to lung development has resulted in a refinement of our understanding of alveolarization. These are the most important refinements:

- Alveoli have an irregular shape that is a result of its space-filling arrangement (Fig. 7) (Mund et al. 2008; Tsuda et al. 2008). Therefore, they do not represent 2/3 of a ball as shown in many text books (Netter 2014). This insight is very important for the alveolar ventilation and deposition of particles, because the 3D microstructure has a significant influence on these two processes, especially at the entrance of the acini (Fishler et al. 2015; Henry et al. 2012; Sznitman et al. 2010). In addition, the irregular structure of the alveoli most likely contributes to an irregular distribution of the strain inside the alveolar septa during breathing and mechanical ventilation (Rausch et al. 2011).

**Fig. 8:**
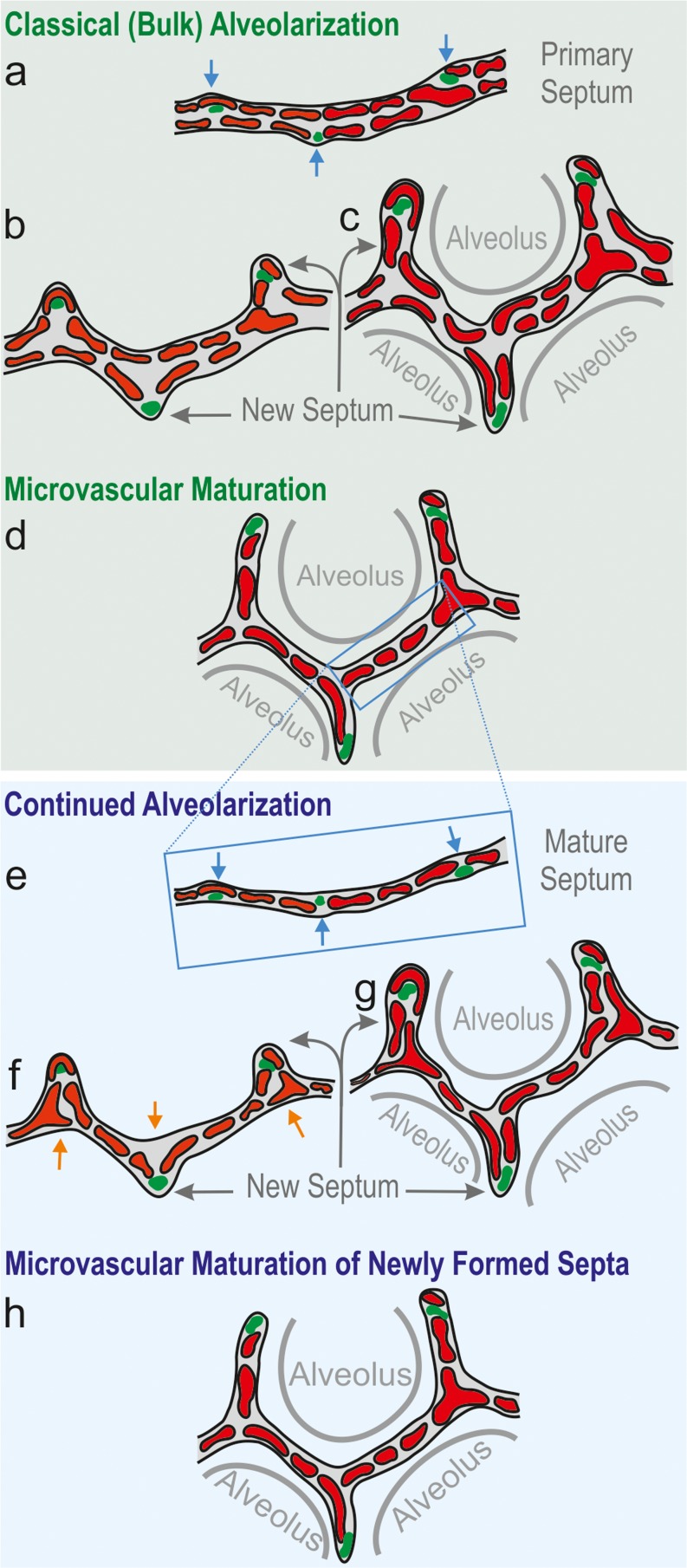
Schematic of classical and continued alveolarization. The interairspace walls present in the saccular stage represent the primary septa. They contain a double-layered immature capillary network (**a**). Each layer appears as a perforated sheet of capillaries (see also Fig. 9). Smooth muscle cell precursors, elastic fibers and collagen fibrils (*green spots*) accumulate at sites where new septa (or secondary septa) will be formed (*blue arrows*, **a**). The secondary septa form by an upfolding (*blue arrows*) of one of the two capillary layers (*red*, **b**). The resulting newly formed secondary septa (*gray arrows*) subdivide preexisting airspaces and the alveoli are born (**c**). At this stage, most of the septa are immature because they possess two capillary layers. During microvascular maturation, the double-layered capillary network fuses to a single-layered one. As a first approximation, microvascular maturation takes place in parallel to alveolarization. Therefore, a significant fraction of new septa are formed starting from a preexisting mature septum containing only a single-layered capillary network (**e**). Now following the mechanism of continued alveolarization, new alveolar septa are still formed by an upfolding of the capillary layer (*red*, **d**–**f**), even if the alveolar surface opposing the upfolding is now missing its capillaries (**d**). This gap is immediately closed by angiogenesis (*orange arrows* in **e **, **f**). In both modes of alveolarization, a sheetlike capillary layer folds up (**b **, **e**) in order to form a double-layered capillary network inside the newly formed septum (**c**, **f**). Regardless of how and when a new septum is formed, it will mature shortly after by a fusion of the double-layered capillary network. (Altered and extended from Burri 1997; Burri 1999; Schittny and Mund 2008; Woods and Schittny 2016, by courtesy of Cambridge University Press, New York)

**Fig. 9:**
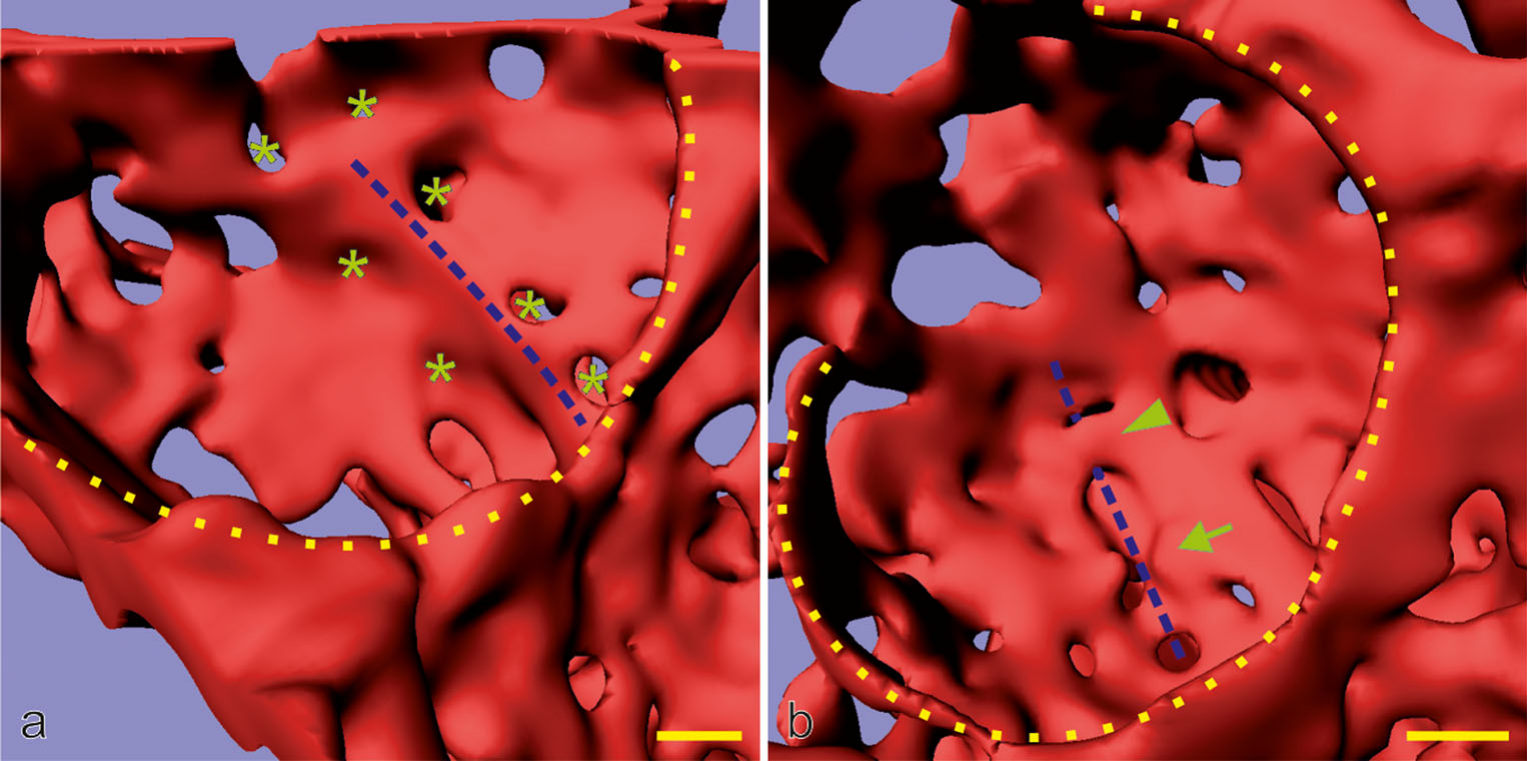
Late alveolarization. Synchrotron radiation-based X-ray tomographic microscopy was used for a 3D visualization of vascular casts (Vascular Mercox^®^) of 21-day-old rat lungs. The lumen of the capillaries are shown, which is identical to their inner surface. Inside the cavity of an alveolus, an upfolding of a single-layered capillary network is shown (*blue dashed lines* in **a**). These kinds of upfoldings are viewed as the formation of new septa. The tomographic dataset permitted to view the backside of the same septum (**b**). A local duplication of the existing capillary network was detected at the basis of the up-folding (covering of the *blue dashed line* in **b**). While parts of the duplication are already formed (*arrowhead*), the remaining duplication is just forming, most likely by sprouting angiogenesis (*arrow* in **b**). Furthermore, (forming) holes in the vascular cast (*green asterisk*) are indicative of intussusceptive angiogenesis (Caduff et al. 1986), which is necessary for the enlargement of the capillary layer as the new septum folds up. The entrances of the alveoli are labeled with a *yellow dotted line*. *Bar* 10 μm (the magnification varies inside the image due to the foreshortened view). (From Schittny et al. 2008 and by courtesy of Springer, Heidelberg)
- A reexamination of microvascular maturation (Roth-Kleiner et al. 2005; and own unpublished results, Figs. 10, 11) revealed that the maturation of the capillary layers of the alveolar septa takes place roughly in parallel to alveolarization. However, the bulk of the maturation occurs mostly during the originally proposed period (Burri 1975; Caduff et al. 1986).

**Fig. 10:**
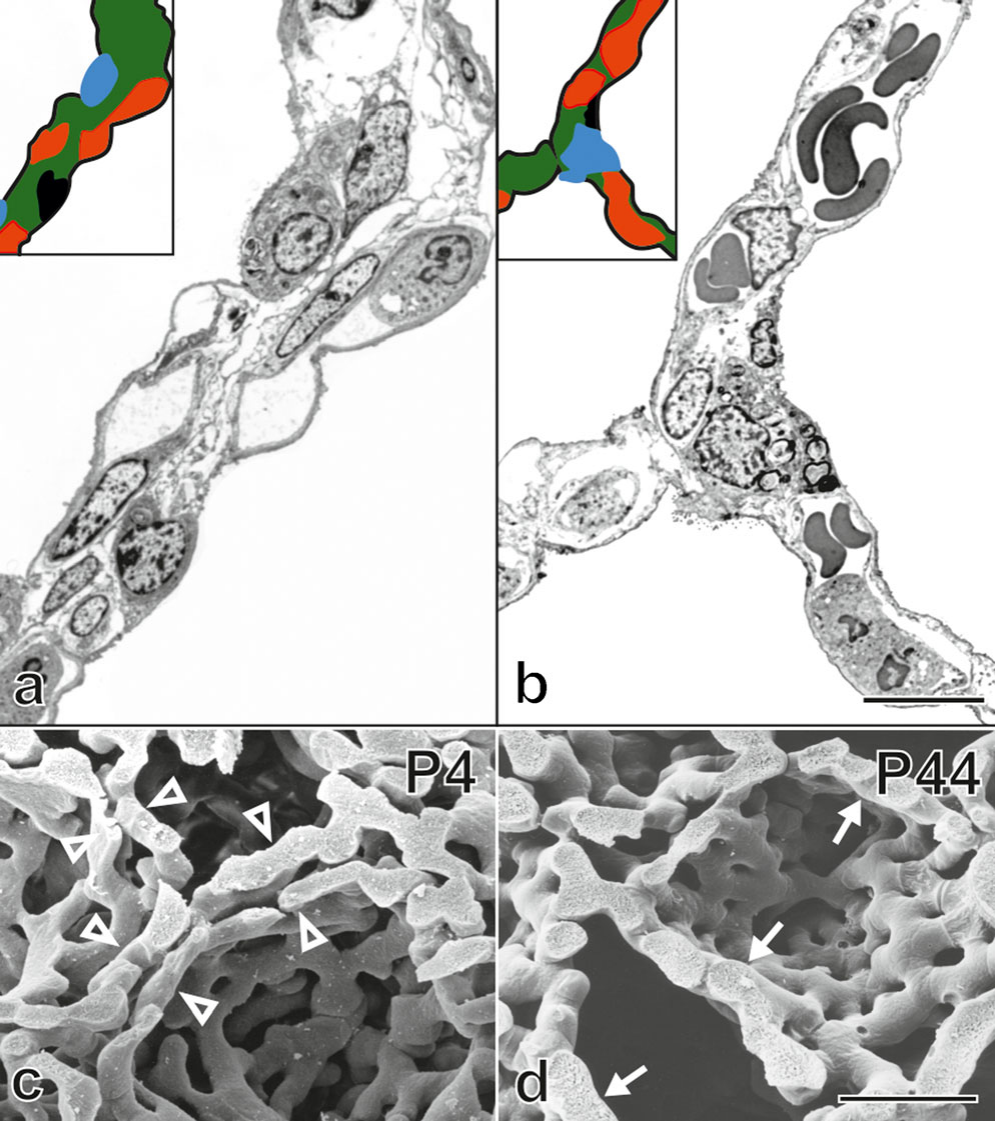
Maturation of the alveolar septa/microvascular maturation. At the beginning of alveolarization, each alveolar surface of an immature septum is served by its own sheetlike capillary layer (**a**, human lung, postnatal day 26, transmission electron micrograph, capillaries in *red*). The two capillary layers are separated by a central sheet of interstitial tissue (*green*). Upon maturation, the two layers of the capillary network start to fuse and the central connective tissue (*green*) is reduced to a septum interwoven with the single-layered capillary network (**b**, adult human lung). This process is even better visible in scanning electron micrographs of vascular cast (Mercox^®^) of rat lungs. While at the start of alveolarization (**c**, day 4) a double-layered capillary network is present (*open arrowhead*), towards the end of alveolarization (**d**, day 44) an optimized single layered capillary network remains. **a **,  **b** schematic: *red* capillaries; *green* interstitial tissue; *black* alveolar epithelial cell type I; *blue* alveolar epithelial cell type II. *Bars* (**a **, **b**,) 10 μm; (**c**, **d **) 25 μm. (From Schittny 2014; Woods and Schittny 2016, by courtesy of Cambridge University Press, New York)

**Fig. 11:**
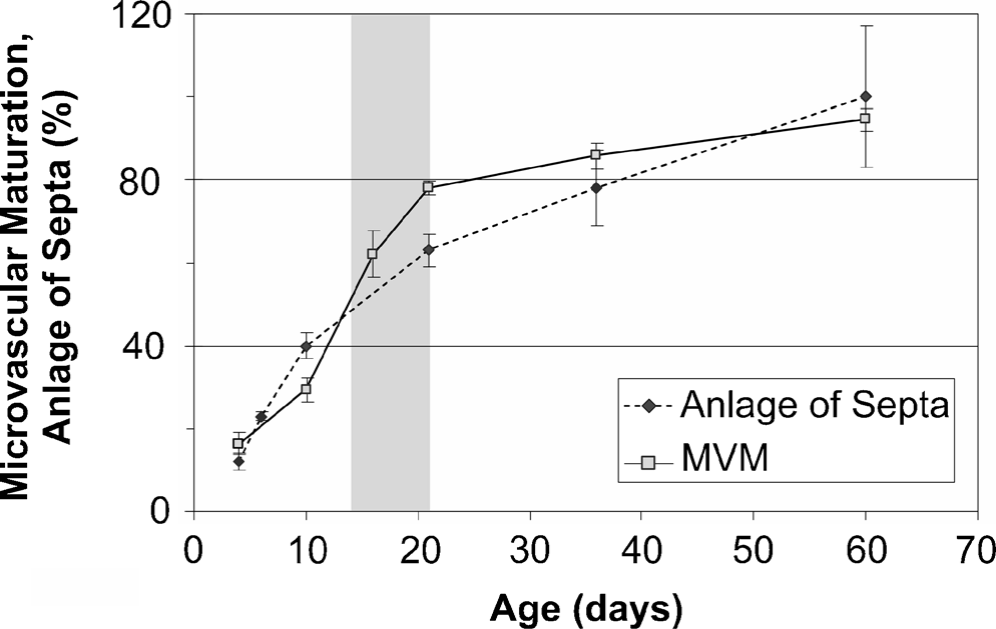
Comparison of alveolarization and microvascular maturation in rats. The anlage of new septa (*dotted line*) increases steadily but with a decreasing slope, meaning that at the beginning of alveolarization a higher speed of the formation of new septa was observed as compared to later days (*dotted line*, *filled circles*). Based mainly on morphological observations, microvascular maturation was originally defined as a phase following alveolarization. In rats, the stage of microvascular maturation was defined as postnatal days 14–21 (period labeled in *gray*) but with a large overlap with the stage of alveolarization (Burri 1975). A stereological estimation of microvascular maturation showed that it starts in parallel with alveolarization and levels off until alveolarization ceases at 95% of maturation (*solid line*, *open square*). The anlage of new septa was calculated based on the estimation of the length of free septal edge. Day 60 was defined as 100% (Schittny et al. 2008). Microvascular maturation was calculated based on the estimation of the alveolar surface area overlaying a single- (mature) or double-layered (immature) capillary network (Roth-Kleiner et al. 2005). Data from Roth-Kleiner et al. (2005) and Schittny et al. (2008) and own unpublished data

### Classical alveolarization (week 36–∼3 years)

At the end of the saccular stage, the lung parenchyma possesses thick, immature septa that contain a double-layered capillary network. Both capillary layers are separated by a core of connective tissue (Figs. 8a, 10a, c). During classical alveolarization, new septa are lifted off immature preexisting septa and will divide the existing airspaces. The upfoldings are visible in 3D images of the lung parenchyma first as low and later as fully grown septa (Fig. 7) and are called secondary septa in order to distinguish them from the primary septa formed by differential growth after branching morphogenesis. The upfoldings appear at locations where smooth muscle cell (precursors) laid down a string of elastic fibers and collagen fibrils (Burri 1974; Lindahl et al. 1997). In addition, tenascin-C is highly expressed at this location (Roth-Kleiner et al. 2014) (Fig. 8a). During folding, the “strings” stay close to the free septal edge of the new septum. The capillary layer facing the airspace takes part in the folding process and the free edge of its folding stays in close contact to the “string” (Fig. 8b). The additional surface area of the capillary network is most likely formed by intussusceptive angiogenesis (Caduff et al. 1986). After the formation of the new septum is initiated, the septum rises up to its full height and the first alveoli are formed (Fig. 8c). In humans (Herring et al. 2014), rhesus monkey (Hyde et al. 2007), rat (Schittny et al. 2008; Tschanz et al. 2014) and mice (Mund et al. 2008), alveolarization shows a bi-phasic behavior. Classical alveolarization starts as a burst and reduces its speed during the switch to continued alveolarization (see below).

### Microvascular maturation (week 36–young adulthood)

Immature primary and secondary septa contain a double-layered capillary network (Fig. 10a, c). These immature septa are rather inefficient in terms of the building material needed for gas exchange. During microvascular maturation, the septa are thinning and the two capillary layers fuse in order to form a more efficient single-layered capillary network in a thin septum (Fig. 10b, d) (Burri 1975; Caduff et al. 1986). This process includes multi-focal fusing of capillary segments and differential growth of the mature single-layered capillary network (Roth-Kleiner et al. 2005; Schittny and Burri 2008). In addition, the interstitial tissue volume decreases significantly (Burri 1974; Kauffman et al. 1974) and a peak of apoptosis was observed during the transition from classical to continued alveolarization (Schittny et al. 1998). The septum of connective tissue is now interwoven with the single-layered capillary network (Fig. 10b).

Originally, the stage of microvascular maturation was described as a stage following the stage of alveolarization, including a large overlap between these two stages (Burri 1975; Caduff et al. 1986). Recently, it was recognized that alveolarization continues until young adulthood (see next section). This new finding triggered a re-examination of the timing of the process of microvascular maturation. Based on stereological estimations done during rat lung development, it turned out that the microvascular maturation takes place in parallel to alveolarization. However, the bulk of maturation happens approximately during the original proposed period (Fig. 11). In order to adapt our view of lung development to these new findings, I would like to propose to recognize the same timing for both processes, meaning that both alveolarization and microvascular maturation start after the saccular stage and continue until young adulthood. This timing takes into account that every newly formed septum first possesses an immature capillary network that will mature shortly afterwards (Fig. 8g, h). Therefore, microvascular maturation has to continue as long as new septa or alveoli are formed. This view is based on rat (Fig. 11) and mice data (own unpublished observations). Stereology-based human or monkey data are not yet available.

Maturation of the pulmonary structural elements does not only include the maturation of the microvasculature but also a maturation of the extracellular matrix including basement membranes. Tissue transglutaminase specifically cross-links proteins by the introduction of γ-glutamyl-ε-lysine-crosslinks. Extracellular tissue transglutaminase stabilizes the extracellular matrix and slows down remodeling. During alveolarization, the appearance of γ-glutamyl-ε-lysine-crosslinks correlates with the maturation of the conducting and respiratory airways including the alveolar septa (Schittny et al. 1997).

### Continued alveolarization (∼2 years–young adulthood)

It was correctly postulated that the formation of a new alveolar septum requires a double-layered capillary network. As a consequence, it was supposed that, after maturation of the microvasculature, no new alveoli may be formed any more (Burri 1975; Caduff et al. 1986). However, in time several cases of alveolarization after microvascular maturation was completed were observed. First, after pneumonectomy, a re-growth of the lung including alveolarization was observed not only in animals (Cagle and Thurlbeck 1988; Hsia et al. 1994) but also in at least one human (Butler et al. 2012). Second, a treatment with glucocorticoids during classical alveolarization strongly decreases the number of alveoli, while a prolonged treatment caused a permanent decrease (Massaro and Massaro 1986) and a short, high-dose treatment caused only a transient one (Tschanz et al. 2003). However, even the permanent alveolar reduction could be rescued by a treatment with retinoic acid after the microvasculature matured (Massaro and Massaro 2000). Third, after starving of small laboratory animals, the number of alveoli was reduced. However, after refeeding, the alveolar number returned to normal (Kalenga et al. 1995a, b). Similar observations were carried out for nutritional emphysema in humans (Coxson et al. 2004). And fourth, studying 3D images of the lung parenchyma, low ridges dividing existing airspaces during rat lung development were observed after maturation of the microvasculature. These rides were indicative of ongoing alveolarization (Fig. 7) (Schittny et al. 2008). At least these four cases triggered a quantitative reinvestigation of alveolarization. Using either the number of alveoli or the length of the free septal edge as a measure, as well as ^3^He-MRI, it was shown for rabbits, rhesus monkey, rats, mice and humans that alveolarization certainly continues until young adulthood, which is later than the originally proposed end of the maturation of the microvasculature (rabbits, Kovar et al. 2002; rhesus monkey, Hyde et al. 2007; rats, Schittny et al. 2008; mice, Mund et al. 2008; and humans, Herring et al. 2014; Narayanan et al. 2012).

Now the classical model of alveolarization had to be refined. Studying high-resolution tomographic images of vascular casts of the alveolar region, a duplication of the single-layered capillary network at sites where the capillary layer folds up to form a new septum was observed by the author (Fig. 9). For very “young” septa where the new septum was just starting to fold up, the duplications of the single-layered capillary network were not yet complete (Fig. 8e, f). A little bit “older” new septa showed complete duplications (Fig. 8g). Therefore, as already postulated in the 1970s (Amy et al. 1977; Burri 1975), a double-layered capillary network represents a prerequisite for the formation of new septa. However, if due to microvascular maturation only one layer is present (Fig. 8e), the required second one will be formed instantly by angiogenesis. As a result, the newly formed septum possesses a folded capillary network or—expressed differently—an immature double-layered capillary network. In a final step, the newly formed septa will mature like any other septum (Fig. 8h) (Schittny et al. 2008).

### Alveolarization of the respiratory bronchioles

Until now, the alveolarization of the respiratory bronchioles have been studied in only one species, the rhesus monkey (Tyler et al. 1988). Alveolarization begins in the most proximal respiratory bronchioles approximately in parallel to the alveolarization of the future alveolar duct and the saccules. It moves distally by time and takes only 5 days in monkeys. The alveoli are formed by an out-pocketing of the future respiratory bronchioles into the surrounding extracellular matrix. They occur on the side opposite to the accompanying pulmonary arteriole. The out-pocketings are first lined with a cuboidal epithelium that later flattens and forms the air–blood barrier (Tyler et al. 1988). The formation of the alveoli of the respiratory bronchioles shows more similarities with the differentiation of the epithelia during the canalicular stage than with the classical or continued alveolarization of the ventilator units. Therefore, it rather resembles the formation of the gas exchanging area of the saccules (primary septa) than of the alveoli (secondary septa).

### Clinical aspects of alveolarization

Meanwhile, it has been shown in many studies (see above) that the lung possesses the ability to form new alveoli as long as it is growing and even during adulthood. Clinically, this potential is very important. First, for lungs thought to be permanently impaired by bronchopulmonary dysplasia, a late recovery has been observed (Carraro et al. 2013). Second, after pneumonectomy or lobe-ectomy, compensatory lung growth including the formation of new alveoli was shown in humans using ^3^HeMRI at least in one case (Butler et al. 2012). In laboratory animals, it was shown that mechanical stretch represents one important factor for this kind of lung recovery (Ravikumar et al. 2013). And third, after treatment of laboratory animals with glucocorticoids, a partial to full recovery of impaired lung development was observed during continued alveolarization. The grade of recovery was dependent on the length of the glucocorticoid treatment (Corroyer et al. 2002; Luyet et al. 2002; Massaro and Massaro 1986; Tschanz et al. 1995, 2003). Recovery could be facilitated in animal experiments by an additional treatment with retinoic acid (Massaro and Massaro 1996; Massaro et al. 2000). However, these experiments also show that the developing lung is susceptible to side effects of a treatment with different drugs including glucocorticoids and retinoic acid derivate.

At a first view, the animal lungs seem to be more adaptive than human lungs. However, while animal studies were done either during or shortly after continued alveolarization was completed, human patients are typically much older than the corresponding age in the animal studies. Therefore, the observed difference between animal studies and clinical experience may be due to age differences. However, regardless of the age of the patient, it is not understood how to re-activate continued alveolarization in order to cure structural lung diseases like pulmonary emphysema and fibrosis, as well as chronic obstructive pulmonary disease (COPD). In principle, it may be possible but our understanding of the molecular mechanism involved is still too limited.

## Conclusion

Even if the first still rudimentary knowledge about the structural lung development goes as far back as Galen (Aelius Galenus von Pergamon, ∼129–216) and Andreas Vesalius (1514–1564), new insights about this topic are currently being obtained. Especially, the newly involved methods of high-resolution 3D structural and functional imaging of the lung are contributing to this. Furthermore, proteomics and gene array methodologies applied down to cellular level will provide new insights into physiological and pathological lung development and function. Combining imaging with molecular analysis has the potential to give us a 3D map of expression patterns and cellular behavior. It is postulated that such maps will enable us to understand regional differences of lung function. The latter is not only a question of lung physiology but also very important for the emerging field of drug delivery via a pulmonary deposition of aerosolized drugs.

**Fig. 12:**
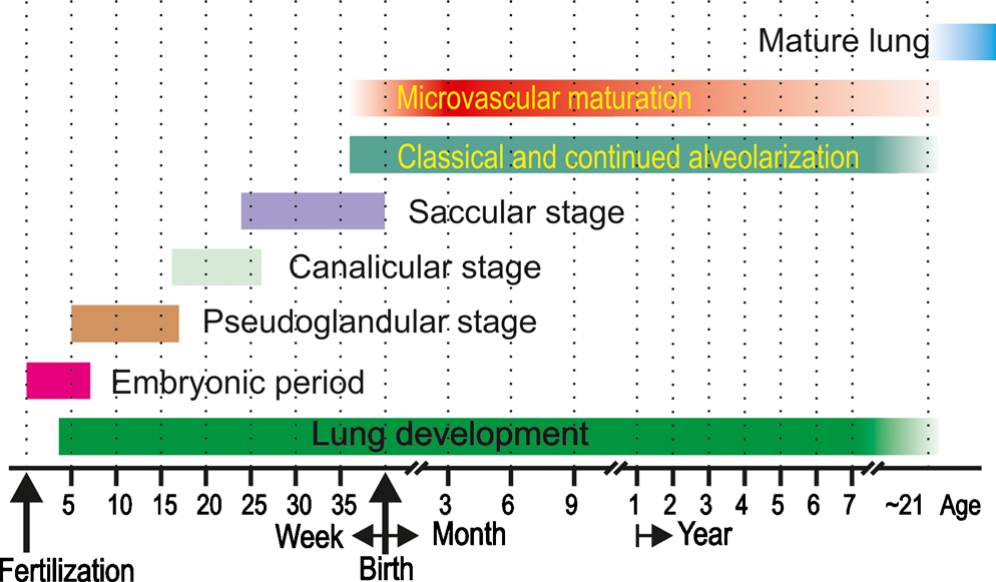
Time scale of human lung development. All stages of lung development are overlapping because most processes of lung development are starting centrally and progress into the periphery. The start and ending of microvascular maturation as well as the end of alveolarization are uncertain. Therefore, the *bars* fade in and out. The embryonic period is not specific for lung development. (Adapted from Schittny 2014 and by courtesy of Springer, Heidelberg)

An updated time scale of human lung development is shown in Fig. 12. However, the different stages of lung development are blending into each other and our definitions of the timing of lung development may not be the most important question in order to achieve a functional lung. For example, the question whether the mechanism of classical versus continued alveolarization is applied at a given time point is less important than the knowledge that the healthy alveolar microvasculature possesses a very high adaptive capacity and will serve any needs. Truly important is the fact that the healthy lung possesses a high capability to adapt, correct and rebuild its structure throughout the entire life. However, diseased lungs like fibrotic and emphysematic lungs lose this capacity. The challenge of the next decade will be to understand why the diseased lungs lose it and how to correct it.
